# Aflibercept Treatment Leads to Vascular Abnormalization of the Choroidal Neovascularization

**DOI:** 10.1155/2018/8595278

**Published:** 2018-04-12

**Authors:** Adam Wylęgała, Filip Wylęgała, Edward Wylęgała

**Affiliations:** ^1^Department of Ophthalmology, District Railway Hospital, Panewnicka 65, 40-765 Katowice, Poland; ^2^School of Medicine with the Division of Dentistry in Zabrze, Medical University of Silesia, Katowice, Poland

## Abstract

Recent studies do not support the hypothesis of vascular normalization in the eyes receiving various types of intravitreous antivascular endothelial growth factor (VEGF). This retrospective study considered 57 eyes of 32 patients with vascular age-related macular degeneration (AMD) undergoing aflibercept treatment. In this study, we measured the vessel density, Horton-Strahler (HS) ramification ratio (complexity), and the length ratio in 14 eyes with choroidal neovascularization treated with 3–5 Eylea injections, 17 eyes receiving 1-2 injections, and 14 treatment-naïve eyes to the use of swept source optical coherence tomography angiography (OCTA). Macular 6 × 6 mm scans were acquired using the DRI OCT Triton by a single trained technician. OCTA images were standardized, binarized, and skeletonized using ImageJ. Then, the HS analysis of the CNV was performed. Our data suggest that the vascular density significantly decreases after an anti-VEGF injection 36 and 93 versus 41 and 87 in treatment-naïve patients. Moreover, CNV before the treatment and in a group with 3–5 injections was more complex than after receiving 1-2 injections. The branch length was not changed. Repeated anti-VEGF can lead to vascular abnormalization and further research is needed to confirm the results of this study.

## 1. Introduction

For more than 50 years, fluorescein angiography has been considered a gold standard for visualizing choroidal neovascularization (CNV); however, this method has many drawbacks such as high camera cost, time-consuming process, and rarely, life-threatening allergic reactions [1].

Since 2012, optical coherence tomography angiography (OCTA) has allowed the visualization of retinal vasculature without the need to inject dye [2]. This technique is useful for several retinal disorders, particularly for imaging CVN [3].

**Figure 1:**
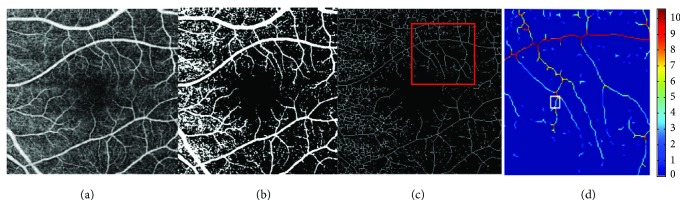
HS analysis of vessel branching in healthy eye. (a) Scan of the funds. (b) Binarized scan. (c) Skeletonized scan: the red frame shows a piece randomly selected for analyses. (d) Heat map image: branches are color-coded by their HS numbers.

The neovascular form of AMD is associated with CNV, in which pathologic new blood vessels grow from the choroid and penetrate through the Bruch membrane into the subretinal pigment in the epithelial and avascular space. CNV can result in hemorrhage, fluid and lipid deposition, pigment epithelial detachment, and scaring that leads to vision loss [4]. Recent studies do not support the hypothesis of vascular normalization in the eyes receiving intravitreous antivascular endothelial growth factor (VEGF). Strahler's analysis was first introduced to determine the river geomorphology by Horton in 1945 and later enhanced by Strahler [5–7]. It is a numerical procedure that sums the branching complexity of mathematical trees such as vascular networks. In this technique, every branch is assigned with Horton-Strahler (HS) number, depending on how many further branches it has. If the branch has no further children, then the HS number is 1. Once the branch has one child with *k* number of branches and other children with *n* branches where *n* = *k* − 1, then HS number of the mother branch is *k*. However, when the mother branch has two or more children with *k* number of branches, then the HS number of mother branch is *k* + 1. This algorithm requires a skeletonized image, in which all terminal branches can be eliminated from the main trunk and analyzed (Figure 1) [8]. We wanted to test whether the anti-VEGF treatment affects the biometric parameters and complexity of the CNV of the retina.

## 2. Material and Methods

The Bioethical Committee at the Medical University of Silesia in Katowice approved the research project in October 08, 2013 (resolution number KNW/0022/KB1/105/13).

This study was conducted in 2017 at the Department of Ophthalmology, Railway Hospital, Katowice, Poland. The patients provided signed consent to have their medical results reviewed for scientific purposes.

The mean age of the 20 women and 12 men considered in the study was 71 ± 13.8 years. All patients underwent a full ophthalmological examination (visual acuity, lens opacity, intraocular pressure, and fundus examination).

During a routine monitoring visit, a single trained technician took a photograph of the fundus of the patients using DRI OCT Triton (Topcon, Tokyo, Japan). This device utilizes a swept source technology with a central wavelength of 1050 nm and can capture more than 100,000 A scans/second. Contrary to AngioVue, that uses split spectrum amplitude decorrelation, Triton uses intensity ratio analysis, rather than splitting the spectrum into narrower bands as in AngioVue [1].

Each patient underwent two 6 mm × 6 mm scans. After quality check, the better image of the CNV located in outer retinal layers was selected.

For HS analysis, the patients were divided into three groups: patients with eyes with AMD naïve to treatment and AMD patients who had already received 1-2 and 3–5 injections of aflibercept (Eylea, Bayer AG, Leverkusen, Germany). For the density analysis, control group of healthy eyes was added. In controls, we measured background of the outer retinal layer, as there was no CNV. Patients who underwent photodynamic therapy and other forms of CNV treatment along with being administered with other intravitreal injections were excluded from the study. Patients with retinal changes secondary to another retinal pathology were also excluded from the study.

Photographs were exported to IMAGEnet 6 and anonymized. In order to reduce projection artifacts, the images were turned into 8-bit gray-scale pictures and binarized. Furthermore, all pictures were skeletonized using the same program.

Then, a HS analysis of branch lengths and ramifications ratio using a semiautomatic algorithm was performed. Ramification ratio is a value describing the unbalanced complexity of a tree *n*_*i*_/*n*_*i*+1_, where *n* is a number of node with HS *i* number. It means that ramification ratio describes complexity of a mathematical tree.

The analysis was performed in ImageJ v151n with the NeuronJ plugin. This program allowed us to calculate the contrast that could be interpreted as the vascular density as well as to perform HS analysis [9]. Furthermore, vascular density was measured as part of the internal control of the results and for comparison with the findings of other studies.

A statistical significance of *p* ≤ 0.05 was applied; Statistica v10.2.1 (StatSoft, Kraków, Poland) was used in all calculations.

Data normality was evaluated using the Shapiro-Wilk tests. Because ramification and HS variables did not pass normality tests, a Kruskal-Wallis nonparametric ANOVA test was used. For measurement of vascular density, one-way ANOVA test with post hoc Fisher LSD test was utilized. Post hoc tests were calculated versus control.

## 3. Results and Discussion

**Figure 2:**
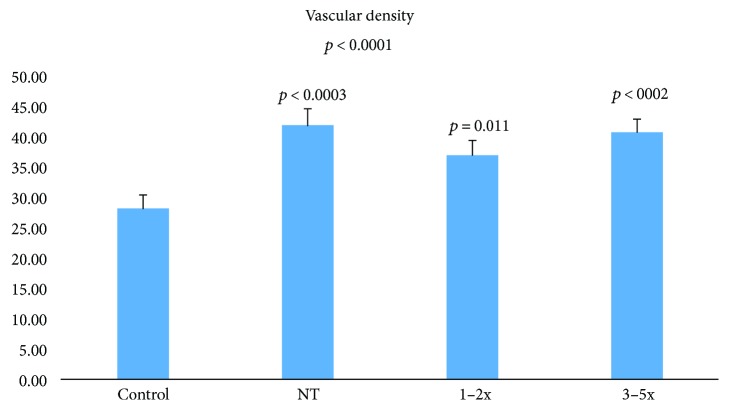
Changes in CNV vascular density during aflibercept therapy. *p* value versus control. HS analysis.

After quality check, we could only include 57 images in our study. Measurements of the vascular density taken before the treatment as well as after receiving 1-2 and 3–5 aflibercept injections and of the healthy controls showed that starting with the very first month following the procedure, the density of the CNV tended to be below the point of naïve treated of 36 and 93 (arbitrary units) versus 41 and 87 for untreated patients (Figure 2). Later, the vascular density rose back again in patients who received more than 3 injections to 40 and 69 which is almost equal to NT. The vascular density of the healthy controls was 28 and 15. The results were statistically significant.

**Figure 3:**
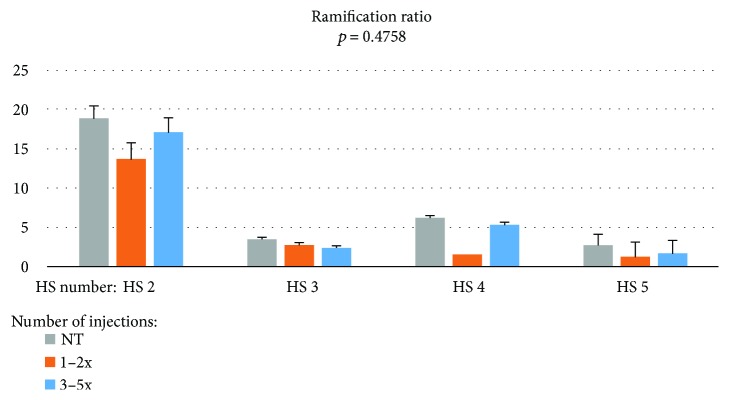
Ramification ratio of the CNV branches during aflibercept treatment.

A comparison of the group of patients with those receiving treatment showed that the statistically insignificant differences between the ramification ratio were the highest in eye naïve to treatment in all branches (Figure 3). This can mean that the CNV before treatment is more complex than in treated patients.

**Figure 4:**
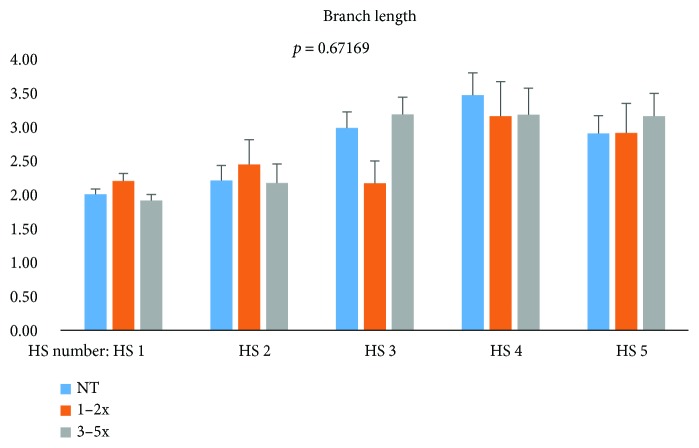
Branch length of the CNV during aflibercept treatment.

Branch length was also assessed (Figure 4); however, the data shows that there is no statistically significant change in branch length during the aflibercept treatment.

**Figure 5:**
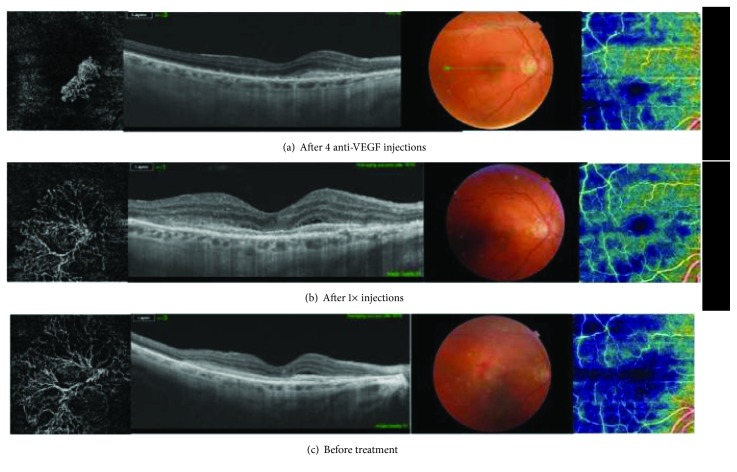
The effects of aflibercept treatment on CNV. Images composed of color and red-free fundus photos. OCTA density map, B scan, and visualization of CNV in outer retina. During treatment, lesion is smaller; however, it becomes more dense.

Our study shows that anti-VEGF treatment with 3 to 5 injections leads to an increase in vascular density of the CNV. The changes in the length of the vessel as well as a general increase in the CNV complexity were observed (Figure 5), but these changes are not statistically significant.

Some studies have reported a lack of normalization after the anti-VEGF treatment.

Spaide [10] was the first author who observed in the OCTA, that receiving recurrent periodic anti-VEGF treatment leads to vascular abnormalization. The author hypothesized that vascular changes of the vessels might be explained by the recurrent pruning of vascular sprouts by VEGF withdrawal during unimpeded arteriogenesis [10]. Lumbroso et al. observed the pruning of the small vessel after each anti-VEGF injection. As a result, the authors observed the strengthening of the trunk vessel and an increase in the blood flow [11].

Spaide advocates that the application of anti-VEGF agents can result in a regression of the new vessels, which can lead to increased vascular resistance and vessel size [10]. Sulzbacher et al. observed an increase in vascular density after VEGF treatment and that associated with a relatively large steam diameter [12]. Some studies also suggested an increased flow in the main vessels after anti-VEGF treatment, which correlates with a diminished branch density [13].

Anti-VEGF treatment results in changes in the CNV lesion after the first injection. Huang et al. observed a diminished CNV flow in the first 2 weeks followed by reappearance of vessels at 4 weeks posttreatment that led to fluid accumulation [14]. To the best of our knowledge, thus far, HS analysis has not been used to assess OCTA images of the retinal vasculature. But, this method is extensively used in geological science and has been used to determine hemodynamic heterogeneity in microvascular networks [5, 15].

One of the biggest limitations of OCTA is the presence of artifacts [16] as well as the need for the patients to fixate for a long time. This implied that patients with poor visual acuity resulting from retinal changes could not be included in the study. We understand that our results of HS analysis might have been disturbed by the presence of artifacts. With the inevitable advance in technology, we believe that sophisticated mathematical analysis might be a new method to quantitatively assess changes.

## 4. Conclusion

We observed a statistically significant change in the vessel density and not significant changes in ramification ratios, which could be a result of vascular abnormalization.

## References

[1] Wylęgała A., Teper S., Dobrowolski D., Wylęgała E. (2016). Optical coherence angiography: a review. *Medicine*.

[2] Ruminski D., Bukowska D., Gorczynska I., Szkulmowski M., Wojtkowski M. Angiogram visualization and total velocity blood flow assessment based on intensity information analysis of OCT data.

[3] de Carlo T. E., Romano A., Waheed N. K., Duker J. S. (2015). A review of optical coherence tomography angiography (OCTA). *International Journal of Retina and Vitreous*.

[4] Liu L., Gao S. S., Bailey S. T., Huang D., Li D., Jia Y. (2015). Automated choroidal neovascularization detection algorithm for optical coherence tomography angiography. *Biomedical Optics Express*.

[5] Horton R. E. (1945). Erosional development of streams and their drainage basins; hydrophysical approach to quantitative morphology. *Geological Society of America Bulletin*.

[6] Strahler A. N. (1957). Quantitative analysis of watershed geomorphology. *Transactions of the American Geophysical Union*.

[7] Schindelin J., Arganda-Carreras I., Frise E. (2012). Fiji: an open-source platform for biological-image analysis. *Nature Methods*.

[8] Ferreira T. (2016). Strahler Analysis. http://imagej.net/.

[9] Schindelin J., Rueden C. T., Hiner M. C., Eliceiri K. W. (2015). The ImageJ ecosystem: an open platform for biomedical image analysis. *Molecular Reproduction and Development*.

[10] Spaide R. F. (2015). Optical coherence tomography angiography signs of vascular abnormalization with antiangiogenic therapy for choroidal neovascularization. *American Journal of Ophthalmology*.

[11] Lumbroso B., Rispoli M., Savastano M. C., Jia Y., Tan O., Huang D. (2016). Optical coherence tomography angiography study of choroidal neovascularization early response after treatment. *Developments in Ophthalmology*.

[12] Sulzbacher F., Pollreisz A., Kaider A. (2017). Identification and clinical role of choroidal neovascularization characteristics based on optical coherence tomography angiography. *Acta Ophthalmologica*.

[13] Lumbroso B., Rispoli M., Savastano M. C. (2015). Longitudinal optical coherence tomography–angiography study of type 2 naive choroidal neovascularization early response after treatment. *Retina*.

[14] Huang D., Jia Y., Rispoli M., Tan O., Lumbroso B. (2015). Optical coherence tomography angiography of time course of choroidal neovascularization in response to anti-angiogenic treatment. *Retina*.

[15] Pries A. R., Secomb T. W., Gaehtgens P. (1996). Relationship between structural and hemodynamic heterogeneity in microvascular networks. *American Journal of Physiology-Heart and Circulatory Physiology*.

[16] Spaide R. F., Fujimoto J. G., Waheed N. K. (2015). Image artifacts in optical coherence tomography angiography. *Retina*.

